# New CT-Detected Pulmonary Abnormalities During Breast Cancer Chemotherapy: Frequency, Imaging Features, and Cumulative-Exposure Associations

**DOI:** 10.3390/cancers18172870

**Published:** 2026-09-05

**Authors:** Jingyi Zhao, Xinyu Kong, Tianye Lin, Li Hu, Yingjian He, Zhaoqing Fan, Yang Yang

**Affiliations:** 1 Breast Center, Peking University Cancer Hospital & Institute, Key Laboratory of Carcinogenesis and Translational Research (Ministry of Education), Beijing 100142, China; 2 Familial & Hereditary Cancer Center, Peking University Cancer Hospital & Institute, Key Laboratory of Carcinogenesis and Translational Research (Ministry of Education), Beijing 100142, China; 3 Department of Radiology, Peking University Cancer Hospital & Institute, Key Laboratory of Carcinogenesis and Translational Research (Ministry of Education), Beijing 100142, China

**Keywords:** breast cancer, chemotherapy, pulmonary abnormalities, chest computed tomography, cumulative dose, cyclophosphamide, paclitaxel

## Abstract

**Background**: This study aimed to quantify the frequency and imaging features of new CT-detected pulmonary abnormalities during breast cancer chemotherapy and examine their associations with cumulative cyclophosphamide and paclitaxel exposure. **Methods**: This single-center retrospective study included 223 patients who underwent an on-treatment chest CT under an early-pandemic infection-control policy. New CT-detected pulmonary abnormalities were defined as new findings on this on-treatment CT that were absent on the pretreatment staging CT. Cumulative cyclophosphamide (C) and paclitaxel (T) doses (mg/m^2^) were calculated from treatment cycles before the on-treatment CT and dichotomized at 1800 and 960 mg/m^2^, respectively. Associations were assessed using Firth-penalized logistic regression. **Results**: New CT-detected pulmonary abnormalities were identified in 34/223 patients (15.2%). Among 25 CT-positive cases with sufficiently detailed CT reports, patchy opacities (14/25, 56.0%) and ground-glass opacities (11/25, 44.0%) were the most frequent findings. Compared with patients with low cyclophosphamide and paclitaxel-equivalent exposure, combined exposure categories showed higher odds of the CT-detected outcome. In the Firth model, cyclophosphamide > 1800 mg/m^2^ was associated with the CT-detected outcome (adjusted odds ratio (OR) 2.38, 95% CI 1.06–5.71; *p* = 0.036), whereas paclitaxel-equivalent exposure > 960 mg/m^2^ (OR 2.46, 95% CI 0.86–6.80; *p* = 0.092) and age ≥ 60 years (OR 2.08, 95% CI 0.91–4.62; *p* = 0.083) were not statistically significant. **Conclusions**: New CT-detected pulmonary abnormalities were common during breast cancer chemotherapy (15.2%). Higher cumulative cyclophosphamide exposure was associated with the CT-detected outcome in multivariable analysis. Prospective longitudinal studies are needed to clarify the clinical significance of these abnormalities and their causality.

## Simple Summary

Some pulmonary abnormalities during breast cancer chemotherapy are not accompanied by obvious respiratory symptoms and may therefore be missed. We reviewed 223 patients who underwent a chest CT during treatment under a pandemic infection-control policy and compared those scans with their pretreatment CTs. New pulmonary abnormalities were identified in 34 patients (15.2%). Higher cumulative cyclophosphamide exposure was associated with the CT-defined outcome in multivariable analysis, whereas older age and higher cumulative paclitaxel exposure were not statistically significant after adjustment. Baseline-referenced CT can detect clinically subtle pulmonary abnormalities, but prospective longitudinal studies are needed to determine their clinical significance and separate cumulative dose from treatment duration.

## 1. Introduction

Chemotherapy is a cornerstone of systemic treatment for breast cancer, but cytotoxic agents are an increasingly recognized cause of lung injury. Chemotherapy-related lung injury spans interstitial pneumonitis, organizing pneumonia, diffuse alveolar damage, pulmonary edema and hemorrhage, as well as pleural, airway, and vascular involvement, and it may culminate in progressive fibrosis [1,2,3]. The reported frequency varies with the agent, the population, and definitions; drug-induced interstitial lung disease is clinically heterogeneous and may be severe or fatal [4,5]. Mechanistically, injury may combine direct dose-related damage to alveolar epithelial and capillary endothelial cells with oxidative and inflammatory pathways [6].

The principal diagnostic challenge is that chemotherapy-related lung injury has non-specific clinical and radiologic features and therefore requires the exclusion of alternative causes, including infection, tumor progression or lymphangitic spread, cardiogenic pulmonary edema, radiation-related injury, and other drug-related pneumonitides [7,8,9]. High-resolution CT can characterize patterns but cannot by itself establish causality, and radiologic–pathologic overlap is substantial [10,11,12,13,14]. The overlap between treatment-related injury and COVID-19-era ground-glass opacities further complicates clinical interpretation [15,16]. Ascertainment against an individual pretreatment CT is therefore important when the objective is to identify treatment-emergent abnormalities.

Pre-existing interstitial lung disease or pulmonary fibrosis is among the most consistent risk amplifiers [17], and cyclophosphamide has been linked to early reversible pneumonitis and late progressive fibrosis [18]. Yet much of this evidence derives from lung cancer cohorts, immunotherapy-containing regimens, or single case reports, and standardized diagnostic criteria and drug-specific, dose-stratified risk estimates are lacking. In breast cancer, reports describe interstitial lung disease associated with docetaxel and paclitaxel [19,20,21]. Moreover, prior work on treatment-related lung injury in breast cancer has focused mainly on radiotherapy: symptomatic radiation pneumonitis has been reported after breast-conserving treatment and may be more frequent with concomitant paclitaxel-containing chemotherapy [22,23]. However, the frequency and imaging patterns of new pulmonary abnormalities arising during chemotherapy alone, as well as their relationship with cumulative drug exposure, are less well defined.

To address these gaps, we conducted a single-center retrospective study of 223 patients with breast cancer who underwent an on-treatment chest CT during the early COVID-19 period. We defined the study endpoint as a new CT-detected pulmonary abnormality relative to the pretreatment staging CT and examined its imaging features and association with cumulative cyclophosphamide and paclitaxel-equivalent exposure.

## 2. Materials and Methods

### 2.1. Patients

This single-center retrospective study analyzed 223 patients with breast cancer who were admitted for chemotherapy at Peking University Cancer Hospital between 5 February and 15 May 2020. Patients were 25–75 years old, had stage I–III disease, and had not received radiotherapy before the on-treatment CT. Molecular subtypes were classified according to estrogen receptor (ER), progesterone receptor (PR), and HER2 status.

Every patient had a pretreatment staging chest CT obtained at diagnosis. Under the pandemic infection-control policy, a non-contrast chest CT was mandatory at the first in-hospital chemotherapy admission. This single on-treatment CT, compared with the pretreatment staging CT, formed the basis for outcome ascertainment. Because CT timing was determined by admission rather than by a fixed treatment milestone, the number of chemotherapy cycles and cumulative exposure at CT varied among patients. The intervals from chemotherapy initiation and from the most recent chemotherapy administration to the on-treatment CT were calculated in days. Subsequent CT examinations were not used to define the primary outcome. Patients with prior chemotherapy or without an eligible baseline comparison were excluded.

### 2.2. Chemotherapy Regimens

Patients received various standard breast cancer chemotherapy regimens, and the treatment phase at the time of on-treatment chest CT varied among individuals. The chemotherapy cycles completed before CT acquisition were extracted from clinical records, including anthracycline–cyclophosphamide-based regimens (e.g., CEF or dose-dense EC), taxane-containing regimens (weekly paclitaxel, dose-dense paclitaxel, or nab-paclitaxel), and docetaxel–cyclophosphamide regimens. Because chest CT was performed during chemotherapy rather than at a predefined treatment milestone, chemotherapy exposure was evaluated according to the actual cycles received before CT acquisition rather than the planned full-course regimen. Cumulative cyclophosphamide and paclitaxel exposures were calculated according to the chemotherapy cycles completed before CT acquisition. Cyclophosphamide exposure was calculated using the standard dose (600 mg/m^2^) per cycle. Paclitaxel exposure was calculated according to regimen-specific administration schedules, including weekly paclitaxel (80 mg/m^2^ per cycle), dose-dense paclitaxel (175 mg/m^2^ per cycle), and nab-paclitaxel regimens (125 mg/m^2^ weekly, 250 mg/m^2^ biweekly, or 260 mg/m^2^ triweekly). Docetaxel was not included in cumulative paclitaxel exposure analyses. According to routine supportive-care practice, dose-dense regimens were supported with either long-acting G-CSF after chemotherapy or short-acting G-CSF according to the treatment schedule. For three-week regimens, blood counts were checked during the cycle, and short-acting G-CSF was given when clinically indicated for significant myelosuppression. G-CSF use was therefore described as supportive care and was not analyzed as a patient-level exposure variable.

### 2.3. Definitions and Outcome Ascertainment

The primary outcome was a new CT-detected pulmonary abnormality, defined as a qualifying pulmonary finding on the on-treatment CT that was absent on the pretreatment staging CT. Findings already present at baseline were not counted. Imaging findings were categorized according to the principal radiologic patterns described in the CT reports. Ground-glass opacity was defined as increased pulmonary attenuation without obscuration of the underlying bronchial and vascular structures. Patchy opacity was defined as focal or multifocal areas of increased pulmonary attenuation without specific characterization as ground-glass opacity. Other findings, including consolidation, nodular opacity, reticular opacity, and atelectatic change, were recorded separately. 

### 2.4. Chest CT Interpretation

Every chest CT study was double-read as part of routine clinical reporting: a primary report was issued by one of seven reporting radiologists and independently verified by one of six reviewing (senior) radiologists, with discrepancies resolved by the reviewing radiologist. Because this CT was mandated for all admitted patients irrespective of respiratory symptoms, ascertainment did not depend on symptom-triggered imaging, reducing the under-detection of asymptomatic injuries inherent to symptom-based studies. The single on-treatment study was interpreted in comparison with the same patient’s pretreatment staging study to distinguish new (treatment-emergent) from pre-existing findings. Because interpretation was performed during routine clinical care rather than as a dedicated research re-read, the readers were not formally blinded to clinical information, and inter-reader agreement was not quantified. These points were considered limitations of the retrospective design.

### 2.5. Exclusion of Infection and COVID-19

The enrollment window coincided with the early phase of the COVID-19 pandemic in Beijing, during which ground-glass and patchy opacities—findings that also characterize COVID-19 pneumonia—required careful differentiation. Two safeguards addressed this: First, in accordance with hospital and municipal policy, patients underwent mandatory SARS-CoV-2 nucleic acid testing during this period, and any patient with confirmed or suspected COVID-19 was excluded. Second, throughout the pandemic, the reporting radiologists routinely and explicitly annotated any CT features suspicious for COVID-19 in the imaging report; none of the CT-positive cases carried such an annotation, so the absence of a COVID-19 flag reflects that the radiology department did not consider those findings representative of COVID-19. Other possible explanations for new pulmonary abnormalities, including bacterial or opportunistic infection, tumor progression or lymphangitic carcinomatosis, pulmonary edema, radiation pneumonitis, and other drug-related pneumonitides, were considered clinically where relevant. The confirmed PCP case was retained because the primary endpoint was defined by the appearance of a new CT abnormality, and its influence was examined in a separate sensitivity analysis.

### 2.6. Statistical Analysis

Continuous variables were summarized as median (interquartile range [IQR]) and compared between groups using the Mann–Whitney U test. Categorical variables were compared using the χ^2^ test or Fisher’s exact test when expected cell counts were small; an exact test with Monte Carlo estimation was used for sparse multi-category comparisons. Unadjusted odds ratios (ORs) with 95% confidence intervals (CIs) were calculated for cumulative-exposure categories, with a Haldane–Anscombe 0.5 correction when a zero cell was present. The primary multivariable analysis used Firth-penalized logistic regression [24,25] with age ≥ 60 years, cumulative cyclophosphamide > 1800 mg/m^2^, and paclitaxel-equivalent exposure > 960 mg/m^2^ as covariates; the model was limited to three covariates because only 34 events were observed. Sensitivity analyses excluded the patient with confirmed PCP and modeled age as a continuous variable, reported per 10-year increase. The relationships between cumulative drug exposure and time from chemotherapy initiation to CT were assessed using Spearman correlation. An exploratory Firth model additionally included the chemotherapy initiation-to-CT interval, reported per 30-day increase, to examine potential confounding by time on treatment. A two-sided *p* < 0.05 was considered statistically significant. The report was prepared with reference to the STROBE guidance for observational studies [26].

## 3. Results

### 3.1. CT-Detected Pulmonary Abnormalities During Chemotherapy

Overall, 34 of 223 patients (15.2%) had a new CT-detected pulmonary abnormality. Baseline clinical characteristics of the study population are summarized in Table 1. Only a minority of patients presented with clinical symptoms. The most common manifestations were fever and cough.

**Figure 1:**
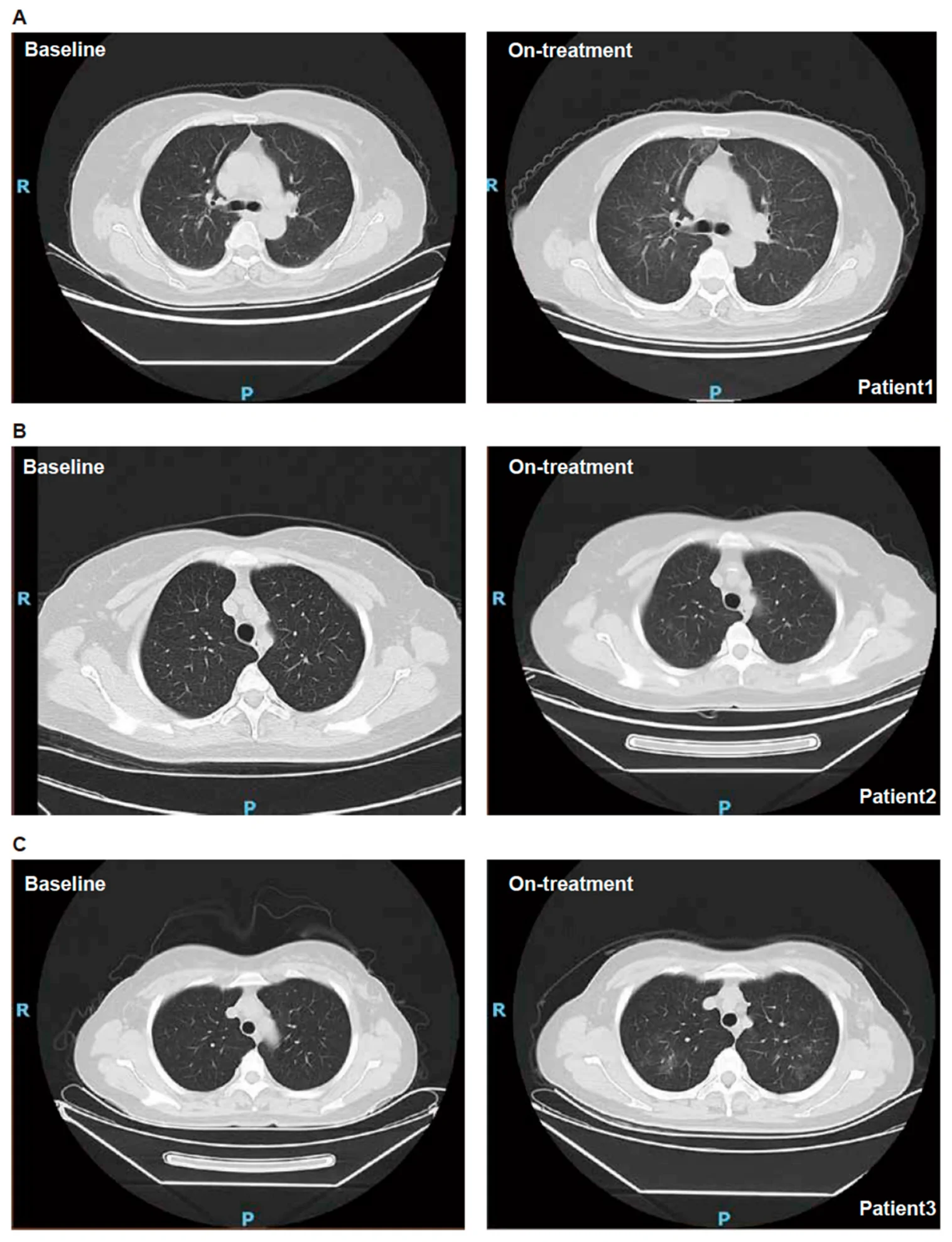
Representative paired baseline and on-treatment CT images. (**A**) Ground-glass opacity and linear/strand-like opacity; (**B**) ground-glass opacity; (**C**) patchy opacity and ground-glass opacity. R, right; P, posterior.

Detailed imaging features could be assessed from the CT report in 25 of the 34 positive cases. Findings were not mutually exclusive. Patchy opacities were present in 14/25 (56.0%) and ground-glass opacities in 11/25 (44.0%). Less frequent findings included linear/strand-like opacity (5/25, 20.0%), nodular opacity (3/25, 12.0%), atelectatic change (2/25, 8.0%), and consolidation (2/25, 8.0%) (Supplementary Table S1). Representative paired baseline and on-treatment CT images are shown in Figure 1.

### 3.2. Clinical and Treatment Characteristics According to CT Status

Clinical characteristics according to CT status are summarized in Table 2, with additional treatment characteristics in Supplementary Table S2. Median age was 52.5 years (IQR 46.0–61.0) in the CT-positive group and 50.0 years (IQR 42.0–57.0) in the CT-negative group (*p* = 0.172). Patients aged ≥ 60 years accounted for 32.4% (11/34) and 21.2% (40/189) of the two groups, respectively (*p* = 0.153).

The cohort was molecularly heterogeneous: 81 patients were HR+/HER2−, 96 were HER2-positive, 42 had TNBC, and 4 had an unknown molecular subtype (Table 1). Among patients with a known subtype, the distribution did not differ between CT-positive and CT-negative groups (*p* = 0.884). Pre-existing pulmonary disease was uncommon (1/34 vs. 1/185 among patients with a known pulmonary history; Fisher *p* = 0.287), and smoking history was also infrequent. The chemotherapy initiation-to-CT interval was 84.0 days (IQR 65.0–107.2) in the CT-positive group and 78.0 days (IQR 41.0–107.0) in the CT-negative group (*p* = 0.151). The corresponding interval from the most recent chemotherapy dose to CT was 1.5 days (IQR 0–10.8) and 0 days (IQR 0–1.0), respectively (*p* < 0.001). The cycle count before CT was 7.5 (IQR 5.0–11.8) versus 6.0 (IQR 3.0–11.0; *p* = 0.098). No significant differences were observed in histological subtype, treatment setting, taxane formulation, docetaxel exposure, or broad regimen exposure pattern (Supplementary Table S2).

### 3.3. Association Between Cumulative Chemotherapy Exposure and CT-Detected Pulmonary Abnormalities

The association between cumulative chemotherapy exposure and CT status is summarized in Table 3. In univariate analysis, patients receiving a cumulative cyclophosphamide dose > 1800 mg/m^2^ showed higher odds of the CT-detected outcome compared with those receiving ≤1800 mg/m^2^, although this association did not reach statistical significance (OR 1.71, 95% CI 0.82–3.59; *p* = 0.152).

Similarly, higher paclitaxel-equivalent cumulative exposure (>960 mg/m^2^) was associated with increased but statistically non-significant odds of the outcome (OR 1.56, 95% CI 0.62–3.93; *p* = 0.347).

When cumulative cyclophosphamide and paclitaxel-equivalent exposure were considered jointly, compared with patients receiving cyclophosphamide ≤ 1800 mg/m^2^ and paclitaxel-equivalent exposure ≤ 960 mg/m^2^, patients with cyclophosphamide ≤ 1800 mg/m^2^ and paclitaxel-equivalent exposure > 960 mg/m^2^ showed increased odds of CT-detected pulmonary abnormalities (OR 3.29, 95% CI 1.05–10.32; *p* = 0.050). Patients with cyclophosphamide > 1800 mg/m^2^ and paclitaxel-equivalent exposure ≤ 960 mg/m^2^ also demonstrated higher odds of the outcome (OR 2.72, 95% CI 1.09–6.78; *p* = 0.036). The subgroup with both higher cumulative cyclophosphamide and paclitaxel-equivalent exposure was limited in size, precluding meaningful interpretation.

### 3.4. Association of Cumulative Chemotherapy Exposure with CT Status After Multivariable Adjustment

To identify factors associated with CT-detected pulmonary abnormalities, a primary multivariable Firth-penalized logistic regression model was constructed, including age ≥ 60 years, cyclophosphamide cumulative dose > 1800 mg/m^2^, and paclitaxel-equivalent cumulative dose > 960 mg/m^2^ (Table 4).

In the adjusted model, cyclophosphamide > 1800 mg/m^2^ was associated with the CT-detected outcome after adjustment for age and paclitaxel-equivalent exposure (adjusted OR 2.38, 95% CI 1.06–5.71; *p* = 0.036). Paclitaxel-equivalent exposure > 960 mg/m^2^ (adjusted OR 2.46, 95% CI 0.86–6.80; *p* = 0.092) and age ≥ 60 years (adjusted OR 2.08, 95% CI 0.91–4.62; *p* = 0.083) were not statistically significant.

In a sensitivity model with age treated as a continuous variable, age had an OR of 1.35 per 10-year increase (95% CI 0.94–1.98; *p* = 0.105), while the cyclophosphamide estimate was 2.41 (95% CI 1.07–5.80; *p* = 0.034) (Supplementary Table S3).

## 4. Discussion

In this cohort, 34 of 223 patients (15.2%) had a new pulmonary abnormality on an on-treatment CT that was not present on the pretreatment examination. Because CT was obtained according to admission and infection-control requirements rather than respiratory symptoms, clinically subtle findings were likely to be captured more often than in symptom-based series [27]. This directly addresses the gap that motivated the study: the frequency, imaging features, and cumulative-exposure associations of CT-detected pulmonary abnormalities during chemotherapy—as opposed to radiotherapy—have rarely been quantified in breast cancer. Ascertainment by imaging rather than by symptoms alone is the central reason our estimate exceeds symptom-based figures; whether this more sensitive detection translates into clinical benefit through earlier intervention cannot be judged here, because patient outcomes beyond the imaging window were not collected, and this practical question remains to be answered in outcome-anchored studies. Within these limitations, the present study provides a CT-based description of treatment-emergent pulmonary abnormalities during breast cancer chemotherapy and their associations with cumulative chemotherapy exposure.

To place these findings in context, the most directly comparable breast cancer literature concerns radiotherapy rather than chemotherapy: radiation pneumonitis occurs in roughly 1% after breast-conserving radiotherapy and rises to about 9–15% when combined with chemotherapy, particularly paclitaxel-containing regimens [22,23]. Our 15.2% cannot be ranked against those figures, however, because the injury mechanism, the population, and above all the ascertainment differ—those estimates rest largely on clinical or symptom-based pneumonitis, whereas our estimate was based on CT obtained regardless of respiratory symptoms, which may partly explain the higher detected frequency relative to symptom-based reports. Against the broader spectrum of drug-induced interstitial lung disease, our estimate sits at the upper end of the 7–21% range cited for high-risk cytotoxic agents [5], but that range spans heterogeneous agents and definitions and likewise permits only qualitative alignment. Finally, the imaging phenotype we observed is consistent with recent descriptions of CT lung injury during breast cancer treatment and with reports of severe drug-induced lung injury during breast cancer chemotherapy in the early pandemic [15,16]. Among the 25 CT-positive patients with sufficiently detailed contemporaneous radiology reports, patchy opacities were described in 14 (56.0%) and ground-glass opacities in 11 (44.0%) (Supplementary Table S1).

The observed association with cumulative cyclophosphamide exposure raises the question of whether cumulative dose itself contributes to pulmonary toxicity or whether it partly reflects longer time on treatment. Cyclophosphamide has long been associated with pulmonary toxicity, including early reversible pneumonitis and late progressive fibrosis [18,28,29,30]. In the present cohort, cumulative cyclophosphamide exposure was weakly correlated with the interval from chemotherapy initiation to CT (Spearman ρ = 0.190, *p* = 0.004), whereas the correlation was stronger for paclitaxel-equivalent exposure (ρ = 0.754, *p* < 0.001). When the initiation-to-CT interval was additionally included in an exploratory Firth model, the cyclophosphamide estimate was attenuated from 2.38 in the primary model to 1.93 (95% CI 0.75–5.13; *p* = 0.173) (Supplementary Table S4). This attenuation is consistent with possible confounding by time on treatment and argues against interpreting the primary association as evidence of a direct dose–response relationship. The subgroup with both higher cumulative cyclophosphamide and paclitaxel-equivalent exposure was also too small for meaningful interpretation.

Several limitations temper these inferences. A first concern is residual confounding by infection: the enrollment window coincided with the early COVID-19 pandemic, during which viral pneumonia posed a diagnostic challenge because of shared ground-glass opacities with drug-induced lung injury. To mitigate this, patients underwent mandatory SARS-CoV-2 testing, and radiologists explicitly flagged any COVID-suspicious features, none of which were present in the cases classified as CT-positive; nevertheless, misclassification of other infection-related opacities cannot be fully excluded and would tend to inflate the detected frequency. Because one patient had microbiologically confirmed Pneumocystis jirovecii pneumonia, we repeated the analysis after excluding this case (Supplementary Table S5). The cyclophosphamide estimate remained in the same direction but was attenuated (adjusted OR 2.24, 95% CI 0.99–5.39; *p* = 0.054), indicating some sensitivity of the result to endpoint classification. A second concern is limited power: with 34 events, multivariable modeling was restricted to three covariates. Smoking and pre-existing pulmonary disease were uncommon, and several regimen categories were sparse, limiting their inclusion in the multivariable model (Supplementary Table S2). Relatedly, the subgroup contrasts in Table 3 were exploratory and not corrected for multiple comparisons, so multiplicity may have amplified chance findings; we therefore interpret them as hypothesis-generating rather than confirmatory. Moreover, chest CT was read through routine clinical double-reading (seven reporting and six reviewing radiologists) rather than a blinded research re-read, so readers were not formally blinded and inter-reader agreement was not quantified. Because ascertainment relied on the single pandemic-mandated CT and later scans were not available, injuries emerging afterward or transient findings resolving before that scan could be missed, and the frequency reflects one time point rather than the whole treatment course. A key missing piece—an individual longitudinal follow-up with a blinded re-read—was not feasible within this retrospective single-center dataset and will be prioritized in future work.

These findings should therefore be interpreted as exploratory. The observed association between higher cumulative cyclophosphamide exposure and CT-detected pulmonary abnormalities requires confirmation in prospective studies with protocolized imaging and more complete assessment of treatment timing and competing causes of pulmonary abnormalities. Future studies using time-varying exposure or landmark approaches may help distinguish a cumulative-dose effect from the influence of treatment duration. Larger cohorts will also be needed to evaluate less common potential risk factors, including pre-existing pulmonary disease.

## 5. Conclusions

New CT-detected pulmonary abnormalities were identified in 15.2% of 223 patients during breast cancer chemotherapy, often without prominent respiratory symptoms. Higher cumulative cyclophosphamide exposure was associated with the outcome in the primary Firth model, but the association was attenuated after exclusion of the confirmed PCP case and after additional adjustment for time on treatment. These findings do not establish a causal dose–toxicity relationship. Prospective longitudinal studies are needed to clarify the clinical significance of treatment-emergent CT abnormalities.

## Figures and Tables

**Table 1:** Baseline characteristics of the study population.

Characteristic	Overall (*n* = 223)
**Age, years**	
median (IQR)	50 (42–58)
**Age category, *n* (%)**	
<60 years	172 (77.1%)
≥60 years	51 (22.9%)
**CT-detected pulmonary abnormality, *n* (%)**	
CT-positive	34 (15.2%)
CT-negative	189 (84.8%)
**Histological subtype, *n* (%)**	
Invasive ductal carcinoma	193 (86.5%)
Invasive lobular carcinoma	7 (3.1%)
Other/mixed subtypes	23 (10.3%)
**Molecular subtype, *n* (%)**	
HR+/HER2−	81 (36.3%)
HER2-positive	96 (43.0%)
TNBC	42 (18.8%)
Unknown	4 (1.8%)
**Previous pulmonary history, *n* (%)**	
Yes	2 (0.9%)
No	217 (97.3%)
Unknown	4 (1.8%)
**Smoking history, *n* (%)**	
Yes	3 (1.3%)
No	220 (98.7%)

Abbreviations: CT, computed tomography; HR, hormone receptor; TNBC, triple-negative breast cancer.

**Table 2:** Clinical characteristics according to CT-detected pulmonary abnormality status.

Characteristic	CT-Positive (*n* = 34)	CT-Negative (*n* = 189)	*p*-Value
**Age, years, median (IQR)**	52.5 (46.0–61.0)	50.0 (42.0–57.0)	0.172
**Age category, *n* (%)**			0.153
<60 years	23 (67.6%)	149 (78.8%)	
≥60 years	11 (32.4%)	40 (21.2%)	
**Molecular subtype, *n* (%)**			0.884 *
HR+/HER2−	11 (32.4%)	70 (37.0%)	
HER2-positive	15 (44.1%)	81 (42.9%)	
TNBC	7 (20.6%)	35 (18.5%)	
Unknown	1 (2.9%)	3 (1.6%)	
**Previous pulmonary history, *n* (%)**			0.287 *
Yes	1 (2.9%)	1 (0.5%)	
No	33 (97.1%)	184 (97.4%)	
Unknown	0	4 (2.1%)	
**Smoking history, *n* (%)**			1.000
Yes	0	3 (1.6%)	
No	34 (100%)	186 (98.4%)	

Abbreviations: CT, computed tomography; HR, hormone receptor; TNBC, triple-negative breast cancer. * Patients with unknown characteristics were excluded from comparison.

**Table 3:** Association between cumulative chemotherapy exposure and CT-detected pulmonary abnormalities.

Characteristic	CT-Positive (*n* = 34)	CT-Negative (*n* = 189)	OR (95% CI)	*p*-Value
**Cyclophosphamide cumulative dose (mg/m^2^)**				
≤1800	14	103	1.00	-
>1800	20	86	1.71 (0.82–3.59)	0.152
**Paclitaxel-equivalent cumulative dose (mg/m^2^)**				
≤960	27	162	1.00	-
>960	7	27	1.56 (0.62–3.93)	0.347
**Combined exposure category (mg/m^2^)**				
C ≤ 1800 + T ≤ 960	7	79	1.00	-
C ≤ 1800 + T > 960	7	24	3.29 (1.05–10.32)	0.050
C > 1800 + T ≤ 960	20	83	2.72 (1.09–6.78)	0.036
C > 1800 + T > 960	0	3	1.51 (0.07–32.18) *	1.000

Abbreviations: C, cyclophosphamide; T, paclitaxel-equivalent exposure; OR, odds ratio; CI, confidence interval. * The Haldane–Anscombe 0.5 correction was applied because the high/high subgroup contained a zero cell; the estimate is imprecise because this subgroup contained only three patients.

**Table 4:** Firth-penalized logistic regression analysis of factors associated with CT-detected pulmonary abnormalities.

Variable	Adjusted OR (95% CI)	*p*-Value
Age ≥ 60 years	2.08 (0.91–4.62)	0.083
Cyclophosphamide cumulative dose > 1800 mg/m^2^	2.38 (1.06–5.71)	0.036
Paclitaxel-equivalent cumulative dose > 960 mg/m^2^	2.46 (0.86–6.80)	0.092

Note: Firth-penalized logistic regression was used because CT-detected pulmonary abnormalities were relatively infrequent and some exposure categories contained sparse observations. This model included age ≥ 60 years, cyclophosphamide cumulative dose > 1800 mg/m^2^, and paclitaxel-equivalent cumulative dose > 960 mg/m^2^.

## Data Availability

The data that support the findings of this study are available from the corresponding author upon reasonable request.

## Supplementary Materials

The following supporting information can be downloaded at https://www.mdpi.com/article/10.3390/cancers18172870/s1, Table S1. Imaging features among CT-positive cases with sufficiently detailed contemporaneous reports; Table S2. Clinical, molecular, and treatment characteristics according to CT-detected pulmonary abnormality status; Table S3. Firth penalized logistic regression with age modeled as a continuous variable; Table S4. (A). Correlation between cumulative exposure and time from chemotherapy initiation to the on-treatment CT; (B). Exploratory Firth model additionally including time from chemotherapy initiation to the on-treatment CT; Table S5. (A). Sensitivity analysis excluding the patient with confirmed Pneumocystis jirovecii pneumonia. (B). Firth-penalized logistic regression analysis of factors associated with CT-detected pulmonary abnormalities excluding the patient with confirmed Pneumocystis jirovecii pneumonia.
