# Supplementary material for: New CT-Detected Pulmonary Abnormalities During Breast Cancer Chemotherapy: Frequency, Imaging Features, and Cumulative-Exposure Associations
Source: Cancers (Basel). 2026 Sep 5;18(17):2870. doi: 10.3390/cancers18172870 (PMC13565309; doi:10.3390/cancers18172870)
Supplement: Supplementary file 1 [file cancers-18-02870-s001.zip › cancers-4505567-supplementary.pdf]

Supplementary Materials

Table S1. Imaging features among CT-positive cases with sufficiently detailed contemporaneous reports.

Imaging features	n/N (%)
Patchy opacity	14/25 (56.0%)
Ground-glass opacity	11/25 (44.0%)
Linear/strand-like opacity	5/25 (20.0%)
Nodular opacity	3/25 (12.0%)
Atelectatic change	2/25 (8.0%)
Consolidation	2/25 (8.0%)

Imaging findings were not mutually exclusive. Feature-level assessment was possible in 25 of the 34 CT-positive patients based on the available radiology reports.

Table S2. Clinical, molecular, and treatment characteristics according to CT-detected pulmonary abnormality status.

Characteristic	CT-positive (n=34)	CT-negative (n=189)	P value
**Age, years, median (IQR)**	52.5 (46.0–61.0)	50.0 (42.0–57.0)	0.172
**Molecular subtype, n (%)**			0.884*
HR+/HER2−	11 (32.4%)	70 (37.0%)	
HER2-positive	15 (44.1%)	81 (42.9%)	
TNBC	7 (20.6%)	35 (18.5%)	
Unknown	1 (2.9%)	3 (1.6%)	
**Histological subtype, n (%)**			0.517†
Invasive ductal carcinoma	31 (91.2%)	162 (85.7%)	
Invasive lobular carcinoma	0	7 (3.7%)	
Other/mixed	3 (8.8%)	20 (10.6%)	
**Treatment setting, n (%)**			0.467†
Neoadjuvant	24 (70.6%)	148 (78.3%)	
Adjuvant	10 (29.4%)	40 (21.2%)	
Both	0	1 (0.5%)	
**Previous pulmonary history, n (%)**			0.287*
Yes	1 (2.9%)	1 (0.5%)	
No	33 (97.1%)	184 (97.4%)	
Unknown	0	4 (2.1%)	
**Smoking history, n (%)**			1.000
Yes	0	3 (1.6%)	
No	34 (100%)	186 (98.4%)	
**Chemotherapy initiation-to-CT interval, days, median (IQR)**	84.0 (65.0–107.2)	78.0 (41.0–107.0)	0.151
**Last chemotherapy dose-to-CT interval, days, median (IQR)**	1.5 (0–10.8)	0 (0–1.0)	<0.001
**Cycle count before CT, median (IQR)**	7.5 (5.0–11.8)	6.0 (3.0–11.0)	0.098
**Taxane formulation before CT, n (%)**			0.288†

None	7 (20.6%)	60 (31.7%)	
Paclitaxel	26 (76.5%)	111 (58.7%)	
Nab-paclitaxel	1 (2.9%)	7 (3.7%)	
Docetaxel	0	8 (4.2%)	
Multiple taxane formulations	0	3 (1.6%)	
**Docetaxel exposure, n (%)**			0.361
Yes	0	9 (4.8%)	
No	34 (100%)	180 (95.2%)	
**Regimen exposure pattern, n (%)**			0.222†
Docetaxel-containing	0	9 (4.8%)	
Cyclophosphamide-containing without paclitaxel-equivalent exposure	7 (20.6%)	60 (31.7%)	
Cyclophosphamide + paclitaxel/nab-paclitaxel	16 (47.1%)	78 (41.3%)	
Paclitaxel/nab-paclitaxel without cyclophosphamide	11 (32.4%)	42 (22.2%)	

Regimen exposure categories were mutually exclusive, with docetaxel-containing regimens classified separately.

* Patients with unknown molecular subtype or pulmonary history were excluded from the corresponding comparison.

† Monte Carlo exact P values based on the Pearson χ^2 statistic were used for sparse multi-category comparisons.

Continuous variables were compared using the Mann–Whitney U test; smoking history, previous pulmonary history, and docetaxel exposure were compared using Fisher's exact test.

No dose reductions, treatment delays, or regimen changes before the index CT were documented.

G-CSF supportive-care practice is described in the Methods and was not analyzed as a patient-level exposure variable.

Table S3. Firth penalized logistic regression with age modeled as a continuous variable.

Variable	Adjusted OR (95% CI)	P value
Age, per 10-year increase	1.35 (0.94–1.98)	0.105
Cyclophosphamide cumulative dose >1800 mg/m²	2.41 (1.07–5.80)	0.034
Paclitaxel-equivalent cumulative dose >960 mg/m²	2.42 (0.84–6.65)	0.098

Table S4A. Correlation between cumulative exposure and time from chemotherapy initiation to the on-treatment CT.

Variable	Spearman ρ	P value
Cyclophosphamide cumulative dose	0.190	0.004
Paclitaxel-equivalent cumulative dose	0.754	<0.001

Table S4B. Exploratory Firth model additionally including time from chemotherapy initiation to the on-treatment CT.

Variable	Adjusted OR (95% CI)	P value
Age ≥60 years	2.08 (0.91–4.62)	0.082
Cyclophosphamide cumulative dose >1800 mg/m²	1.93 (0.75–5.13)	0.173
Paclitaxel-equivalent cumulative dose >960 mg/m²	1.73 (0.48–6.11)	0.395
Chemotherapy initiation-to-CT interval, per 30-day increase	1.13 (0.86–1.45)	0.353

This model was exploratory and was used to assess possible confounding by time on treatment; it was not intended to replace the primary three-covariate model.

Table S5A. Sensitivity analysis excluding the patient with confirmed Pneumocystis jirovecii pneumonia.

Characteristic	CT-positive (n=33)	CT-negative (n=189)	OR (95% CI)	*P* value
**Cyclophosphamide cumulative dose (mg/m²)**				
≤1800	14	103	1.00	-
>1800	19	86	1.63 (0.77–3.43)	0.200
**Paclitaxel-equivalent cumulative dose (mg/m²)**				
≤960	26	162	1.00	-
>960	7	27	1.62 (0.64–4.09)	0.308
**Combined exposure category (mg/m²)**				
C≤1800 + T≤960	7	79	1.00	-
C≤1800 + T>960	7	24	3.29 (1.05-10.32)	0.050
C>1800 + T≤960	19	83	2.58 (1.03-6.48)	0.055
C>1800 + T>960	0	3	1.51 (0.07-32.18)*	1.000

Abbreviation: C, cyclophosphamide; T, paclitaxel-equivalent exposure; OR, odds ratio; CI, confidence interval.
* Haldane–Anscombe 0.5 correction applied because of the zero cell.

Table S5B. Firth-penalized logistic regression analysis of factors associated with CT-detected pulmonary abnormalities excluding the patient with confirmed Pneumocystis jirovecii pneumonia.

Variable	Adjusted OR (95% CI)	*P* value
Age ≥60 years	1.89 (0.80–4.26)	0.141
Cyclophosphamide cumulative dose >1800 mg/m²	2.24 (0.99–5.39)	0.054
Paclitaxel-equivalent cumulative dose >960 mg/m²	2.46 (0.86–6.77)	0.092
